# Identification of *TARDBP* Gly298Ser as a founder mutation for amyotrophic lateral sclerosis in Southern China

**DOI:** 10.1186/s12920-022-01327-4

**Published:** 2022-08-05

**Authors:** Fanxi Xu, Sen Huang, Xu-Ying Li, Jianing Lin, Xiuli Feng, Shu Xie, Zhanjun Wang, Xian Li, Junge Zhu, Hong Lai, Yanming Xu, Xusheng Huang, Xiaoli Yao, Chaodong Wang

**Affiliations:** 1 Department of Neurology, National Clinical Research Center for Geriatric Diseases, Xuanwu Hospital of Capital Medical University, No.45 Changchun Street, Beijing, 100053 China; 2 Department of Neurology, Guangdong Provincial Key Laboratory of Diagnosis and Treatment of Major Neurological Diseases, National Key Clinical Department and Key Discipline of Neurology, The First Affiliated Hospital, Sun Yat-Sen University, Guangzhou, 510080 China; 3 Institute of Genetics and Developmental Biology, Chinese Academy of Sciences, Beijing, China; 4 National Human Genome Center in Beijing, Beijing, China; 5 Department of Neurology, West China Hospital of Sichuan University, Chengdu, China; 6 Department of Neurology of the First Medical Center, Chinese PLA General Hospital, Beijing, China

**Keywords:** Amyotrophic lateral sclerosis, TARDBP, G298S mutation, Founder effect, China

## Abstract

**Background:**

Amyotrophic lateral sclerosis (ALS) is a progressive neurodegenerative disease characterized by predominant impairment of upper and lower motor neurons. Over 50 *TARDBP* mutations have been reported in both familial (FALS) and sporadic ALS (SALS). Some mutations in *TARDBP*, e.g. A382T and G294V, have genetic founder effects in certain geographic regions. However, such prevalence and founder effect have not been reported in Chinese.

**Methods:**

Whole-exome sequencing (WES) was performed in 16 Chinese FALS patients, followed by Sanger sequencing for the *TARDBP* p.Gly298Ser mutation (G298S) in 798 SALS patients and 1,325 controls. Haplotype analysis using microsatellites flanking *TARDBP* was conducted in the G298S-carrying patients and noncarriers. The geographic distribution and phenotypic correlation of the *TARDBP* mutations reported worldwide were reviewed.

**Results:**

WES detected the *TARDBP* G298S mutation in 8 FALS patients, and Sanger sequencing found additional 8 SALS cases, but no controls, carrying this mutation. All the 16 cases came from Southern China, and 7 of these patients shared the 117-286-257-145-246-270 allele for the D1S2736-D1S1151-D1S2667-D1S489-D1S434-D1S2697 markers, which was not found in the 92 non-carrier patients (0/92) (*p* < 0.0001) and 65 age-matched and neurologically normal individuals (0/65) (*p* < 0.0001). The A382T and G298S mutations were prevalent in Europeans and Eastern Asians, respectively. Additionally, carriers for the M337V mutation are dominated by bulbar onset with a long survival, whereas those for G298S are dominated by limb onset with a short survival.

**Conclusions:**

Some prevalent *TARDBP* mutations are distributed in a geographic pattern and related to clinical profiles. *TARDBP* G298S mutation is a founder mutation in the Southern Chinese ALS population.

**Supplementary Information:**

The online version contains supplementary material available at 10.1186/s12920-022-01327-4.

## Introduction

Amyotrophic lateral sclerosis (ALS) is a progressive, fatal neurodegenerative disease characterized by predominant impairment of upper and lower motor neurons, with an incidence of approximately 1–2 per 100,000 people. Patients usually suffer from progressive muscle weakness and atrophy and die of respiratory failure 3–5 years after the onset [1, 2]. The etiology of ALS is not fully understood. Appropriately 90% of patients appear sporadically (sporadic ALS, SALS), while ~ 10% of patients have a family history positive for ALS (familial ALS, FALS) or frontotemporal dementia (FTD). With the advancement of sequencing technologies, rapid increasing number of genes and mutations have been identified in ALS, suggesting that genetic factors play important roles in its pathogenesis.

To date, variants in 25 genes that fulfill adequate criteria for causation for ALS have been reported [3, 4]. Mutations in these genes have been identified in approximately two-thirds of FALS and 10% of SALS cases [5]. Recent studies have identified *SOD1*, *FUS*, *TARDBP* and *C9orf72* as the major ALS-related genes in both European and Asian populations [6]. Mutations in *SOD1* are the most common cause for ALS in the world, and are detected in ~ 20% of FALS and 3% of sporadic ALS (SALS) [7]. *C9orf72* mutation is a prevalent cause for ALS in populations of European ancestry, but was rarely reported in Eastern Asians [8, 9]. *FUS* mutations seem to be the most frequent genetic cause in early-onset sporadic ALS patients [10, 11]. A characteristic feature of degenerating neurons in ALS patients is the presence of cytoplasmic insoluble and ubiquitinated inclusions containing abnormal aggregates of TAR DNA-binding protein (TDP-43), encoded by the *TARDBP* gene [12]. However, it is worthwhile to explore that since the first report of TDP-43 positive aggregates in ALS and FTD, they were also reported in other neurodegenerative diseases as a secondary feature [13]. TDP-43 is involved in RNA processing, splicing and transport. It consists of an N-terminal nuclear localization signal followed by two RNA recognition motifs and a C-terminal glycine rich domain. To date, more than 50 *TARDBP* mutations have been reported which explain approximately 4% of FALS cases and a smaller proportion of FTD cases [14]. Of course, this does not mean that all mutations are actually pathogenic mutations. But, all *TARDBP* pathogenic mutations exhibit an autosomal dominant pattern of inheritance [15]. Most of the disease-associated TARDBP mutations are located within the C-terminal proximal Gly-rich region, indicating critical involvement of this region in the pathogenesis of TDP-43 proteinopathy [16, 17]. Indeed, emerging evidence supports that TDP-43 may act like a prion to initiate cascades of protein misfolding [18].

Some mutations in ALS-associated genes are in high frequency in certain geographic regions and genetic founder effects have been examined for their associations with ALS. The distribution of ALS causing genes varies widely across populations and may differ widely between two seemingly similar countries. Founder mutations for ALS have been established in Italians (*SOD1*: D124G and G41S) [19–21], North American (*SOD1*: A4V) [22], Polish (*SOD1*: L144S) [23], Brazil (*VAPB*: P56S) [24] and Germany (*SOD1*: R115G) [25]. Hexanucleotide (G_4_C_2_) repeat expansion (HRE) in *C9orf72* has a single founder and is the most common mutation in familial and sporadic ALS in Europe, mainly in Finnish [26]. The HRE in *C9orf72* were detected in 46.0% of familial ALS and 21.1% of sporadic ALS in Finnish. While several founder mutations have been reported, by far the most common is the *SOD1* D90A (highly prevalent in Sweden and Finland but are rare in neighboring countries) [27]. In *TARDBP*, the most commonly reported missense mutations are A382T and M337V, and some of the most well-studied mutations are A315T, Q331K, M337V, D169G, G294A/V, Q343R, etc. Moreover, ALS cases carrying the A382T and G295S mutation of *TARDBP* and the *C9orf72* repeat expansion shared distinct haplotypes across these loci in Sardinia [28]. In Chinese ALS cases, H47R, R521H and M337V are the most frequent mutations in *SOD1*, *FUS* and *TARDBP*, respectively [29]. However, there is still no haplotype analysis to support the founder effect of these mutations.

Here, we identified a prevalent heterozygous Gly298Ser mutation (G298S, as follows) in *TARDBP* in both FALS and SALS in Guangdong and Guangxi, two neighboring Southern Chinese provinces. Haplotype analysis with the microsatellites surrounding the gene further confirmed a founder effect of this mutation in Southern, but not Northern Chinese. We also reviewed the global distribution of the mutations in *TARDBP* and revealed geographic distribution patterns and clinical relevance of some prevalent mutations.

## Materials and methods

### Subjects

A total of 798 FALS and SALS cases were recruited between 2016 and 2021, including 462 from The First Affiliated Hospital of Sun Yet-Sen University, 232 from Xuanwu Hospital of Capital Medical University and the Chinese PLA General Hospital, and 104 from West China Hospital of Sichuan University. Among these, 16 had a family history (FALS) and 782 were sporadic ALS (SALS). All the patients were diagnosed as definite, probable or possible ALS by at least 2 neuromuscular specialists, based on the revised EI Escorial criteria (2000) [30]. During the same period, 1325 ethnicity-matched controls without any neurological diseases were enrolled. To avoid recruiting presymptomatic patients, we tended to recruit older people as healthy controls. Therefore, all healthy controls were older than 55 years.

### Ethnics approvals and patient consents

Written informed consent was obtained from all participants, as approved by the Ethics Committee and the Expert Committee of Xuanwu Hospital of Capital Medical University (Clinical Research Audit: [2021] 034) and The China Human Genetic Resource Administration Office (China Ministry of Science and Technology Genetics Audit: [2021] CJ1167).

### Screening of C9orf72 (GGGGCC)n repeat

Two-step polymerase chain reaction (PCR) was performed to detect *C9orf72* GGGGCC hexanucleotide repeat expansion (HRE) as previously described [31]. Briefly, fluorescent fragment-length analysis was performed with genotyping primers. The samples with a homozygous peak pattern were analyzed by fluorescent repeat-primed PCR to identify HREs. The HRE was defined as repeat number > 30 indicated by the typical “saw-tooth” pattern seen by repeat-primed PCR [32].

### Whole-exome sequencing for FALS

As it is easier to find causative mutations in FALS, whole-exome sequencing (WES) was performed in all the 16 FALS cases whose DNA samples were available. The WES was performed as the methods described previously [33]. Briefly, whole blood-derived DNA was captured to generate a sequencing library using the Agilent SureSelect Human All Exon V6 Kit (Agilent Technologies, Santa Clara, CA). The enriched library targeting the exome was sequenced on the HiSeq 2500 platform (Illumina, San Diego, CA) to get paired-end reads with read length of 90 bp. Variants were annotated using the Realigner Target Creator in Genome Analysis Toolkit (GATK) and the ANNOVAR, and aligned to the human genome (GRCh37/hg19), and the public polymorphism (http://www.ncbi.nlm.nih.gov/SNP), Human Gene Mutation (http: //www. hgmd.cf. ac. uk/), ClinVar (https://www.ncbi.nlm.nih.gov/clinvar/) and Pubvar (https://www.pubvar. com) databases. We only selected the pathogenic, likely pathogenic or uncertain significance variants according to the 2015 American College of Medical Genetics and Genomics and the Association for Molecular Pathology (ACMG/AMP) criteria [34]. Mutations in 25 well-known ALS causative genes, including *ALS2, ANG, ANXA11, AR, CHMP2B, DAO, DCTN1, FUS, FIG4, GRN, KIF5A, NEFH, OPTN, PFN1, PRPH, SOD1, SETX, SIGMAR1, TARDBP, TIA1, TFG, TAF15, UBQLN2, VAPB* and VCP, were analyzed [35–38]. The genes were selected for sufficient genetic and functional evidence of gene-disease association. We analyzed both heterozygous and homozygous mutations for these genes. Further Sanger sequencing was used to confirmed the mutation.

### Sanger sequencing for the TARDBP G298S mutation in the SALS patients and controls

In order to investigate whether the *TARDBP* c.892G > A (p.Gly298Ser) mutation was also present in SALS patients and controls, Sanger sequencing of the mutation was performed in 782 sporadic cases (including 511 Southern and 271 Northern) and 1325 controls. The primers were designed by the Primer 3 v.0.4.0 (http://bioinfo.ut.ee/primer3-0.4.0/primer3/) for polymerase chain reaction (PCR): forward primer: 5’-TTGCTTATTTTTCCTCTGGCTTTAGAT-3’; reverse primer: 5’-TACTCCACACTGAACAAACCAATTT-3’. PCR product was purified and sequenced by ABI 3730 DNA analyzer (Applied Biosciences, Inc, CT, USA). The Chromas 2.6.5 software (Technelysium, South Brisbane, Australia) was used for sequence reading. Variant position was based on GenBank accession number NC_000001.10, transcript position was based on NM_007375.3, and protein position based on NP_031401.1, according to the hg19/GRCh37 reference sequence. Furthermore, for the SALS patients screened for carrying the TARDBP G298S mutation, we performed whole-exome sequencing to exclude other mutations. All these WES data from both sporadic cases and familial cases carrying the same mutation were used to determine the degree of relatedness of samples (quantified as the PI_HAT metric) by applying the identity-by-descent algorithm within the PLINK toolset [39].

### Haplotype analysis using the microsatellite markers

To test whether the *TARDBP* G298S mutation carriers share the common ancestry, we performed haplotype analysis for the ALS patients with the mutation using the eight microsatellite markers in 1p36.22 spanning a region of about 7 Mb (D1S450, D1S244, D1S2736, D1S1151, D1S2667, D1S434, D1S489, D1S2697) surrounding the *TARDBP* gene, according to Orrù et al. [40]. They were genotyped in the 16 ALS individuals carrying the G298S mutation and 92 cases not carrying the mutation, using the fluorescent-labeled primers designed and listed in Additional file 1: Table S1. The alleles were typed by electrophoresis on an ABI 3730 (Applied Biosystems, Foster City, CA, USA) and analyzed using GeneMarker Demo software V2.6.3 (SoftGenetics, State College, PA, USA). Haplotype frequencies and association statistics for the markers were constructed using PHASE version 2 software [41].

### Statistical analysis

The results of all continuous data are presented in this report as mean ± standard deviation. The difference in haplotype distribution between mutation carriers and noncarriers was evaluated by χ^2^ and Fisher’s exact tests. The *p* value for statistical significance was set as < 0.05.

## Results

### Demographics and clinical phenotype of the patients

A total of 798 ALS patients were included in this study, including 16 FALS (from 15 pedigrees) and 782 SALS. All patients were of Chinese Han origin. Among the 798 cases diagnosed, 482 were males and 316 were females. The mean age of ALS onset was 52.77 ± 10.40 years. For initial symptoms, 615 patients (77.07%) presented with onset of limb symptoms, while 183 (22.93%) presented with a bulbar-onset. The survival time ranged from 6 to 122 months. In the controls, 749 were males and 576 were females, and the mean age were 69.23 ± 14.62 years.

### Mutations identified in the FALS patients by whole-exome sequencing

**Fig. 1:**
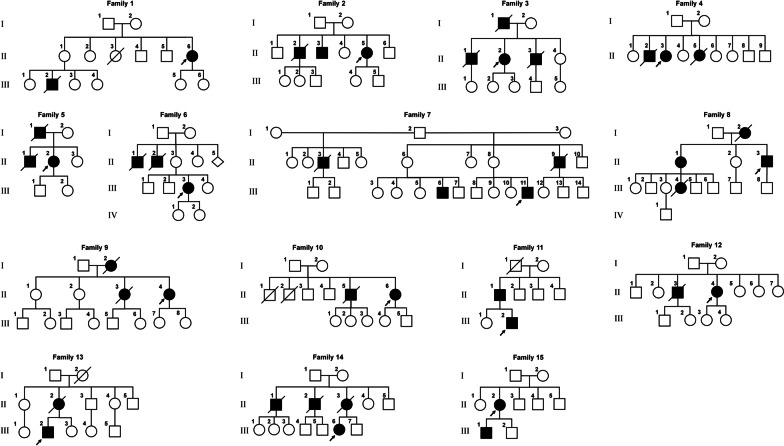
Pedigree map of the16 FALS patients from 15 pedigrees. Pedigrees 1–7: families carried the TARDBP G298S mutation; pedigrees 8–14: families carried other mutations in TARDBP or other genes; pedigree 15: the family with no mutation detected. Black symbols represent patients affected with ALS, white symbols represent unaffected individuals. Arrowheads indicate the probands

In total, we detected 134 variants of all types in the 25 selected genes (*ALS2, ANG, ANXA11, AR, CHMP2B, DAO, DCTN1, FUS, FIG4, GRN, KIF5A, NEFH, OPTN, PFN1, PRPH, SOD1, SETX, SIGMAR1, TARDBP, TIA1, TFG, TAF15, UBQLN2, VAPB* and VCP). After sequential screening and filtering by population frequency (max frequency < 0.01) and selection of variant types (missense, stop gained, frameshift, in-frame and splicing) and functional /conservation prediction (SIFT, CADD, Polyphen2, GERP++), 7 rare damaging variants were identified in the 16 FALS patients. Among all the rare damaging variants, 5 were pathogenic and 2 were likely pathogenic, according to the ACMG/AMP guidelines. Among the 16 patients, 10 carried mutations in *TARDBP* (G298S, N378D and N345K), 3 carried mutations in *SOD1* (C112Y and G142A), 1 carried a *FUS* mutation (R521C) and 1 carried a VCP mutation (R155C), and no mutation was identified in one patient (F15) (Table 1). No pathogenic variants in other genes were identified in the cases carrying the TARDBP G298S mutation. The GGGGCC hexanucleotide repeats in *C9orf72* were 2 to 11 copies, which means that the alleles were not expanded and non-pathogenic. *C9orf72* repeat expansion segregates with disease in the Finnish population as a founder haplotype, underlying 46.0% of familial ALS and 21.1% of sporadic ALS in that population [32]. Strikingly, among all the mutations, G298S was the most frequently (8/16, 50%) identified, and all these 8 patients were from 7 pedigrees in Guangdong Province, a region in Southern China (Fig. 1). This is a high frequency mutation that has not been widely reported.

**Table 1:** Clinical data and mutations identified in the 16 FALS patients

Patient^a^	Gender	Birthplace (provinces)	AAO (y)	Onset site	Duration (months)	Genetic variant	ACMG
**F1**	M	Guangdong	50	Upper limb	48 (alive)	TARDBP, c.892G > A, p. Gly298Ser	P
**F2**	F	Guangdong	50	Lower limb	15	TARDBP, c.892G > A, p. Gly298Ser	P
**F3-1**	F	Guangdong	62	Upper limb	13	TARDBP, c.892G > A, p. Gly298Ser	P
**F3-2**	M	Guangdong	54	Bulbar	20	TARDBP, c.892G > A, p. Gly298Ser	P
**F4**	F	Guangdong	73	Upper limb	10	TARDBP, c.892G > A, p. Gly298Ser	P
**F5**	F	Guangdong	49	Bulbar	11	TARDBP, c.892G > A, p. Gly298Ser	P
**F6**	F	Guangdong	53	Upper limb	20	TARDBP, c.892G > A, p. Gly298Ser	P
**F7**	M	Guangdong	46	Bulbar	14	TARDBP, c.892G > A, p. Gly298Ser	P
F8	F	Guangdong	38	Lower limb	NA	SOD1, c.425G > C, p. Gly142Ala	P
F9	F	Sichuan	43	Bulbar	NA	FUS, c.1561C > T, p. Arg521Cys	P
F10	F	Guangxi	60	Bulbar	NA	TARDBP, c.1035C > A, p. Asn345Lys	LP
F11	M	Guangdong	33	Lower limb	NA	VCP, c.463C > T, p. Arg155Cys	P
F12	F	Hunan	50	Upper limb	NA	SOD1, c.335G > A, p. Cys112Tyr	P
F13	M	Guangdong	40	Upper limb	12 (alive)	TARDBP, c.1132A > G, p. Asn378Asp	LP
F14	F	Hunan	32	Upper limb	NA	SOD1, c.335G > A, p. Cys112Tyr	P
F15	F	Guangdong	66	Upper limb	NA	Not identified	-

*NA* stands for “not available.”, *LP* Likely Pathogenic, *AAO* Age at onset. Patient^a^, The bolded numbers in parentheses represent our FALS patients carrying the G298S mutation. F3-1 and F3-2: two siblings of the family 3 (II-2 and II-3)

### Carriers for TARDBP G298S mutation in the SALS patients

The identified *TARDBP* G298S mutation in FALS patients were extremely rare in the databases (Minor allele frequency = 0; non-neurological component of GnomAD v2.1.1). To further identify the carriers for G298S mutation, we screened it by Sanger sequencing in 782 SALS patients (including 511 Southern and 271 Northern Chinese) and 1,325 ethnically matched healthy controls. In all the cases, 8 were found to carry the *TARDBP* G298S mutation. Ultimately, we found 16 patients carried the same G298S mutation, in which 13 were from Guangdong Province and 3 were from its neighboring province-Guangxi. However, the mutation was not detected in the 232 Northern or 104 Western Chinese ALS patients, neither in the 1325 controls. Given the strong geographic preference of the mutation, we hypothesize that G298S might be a founder mutation in the Guangdong-Guangxi region.

### The TARDBP G298S mutation was associated with an ancestral haplotype

To investigate whether there was genetic relatedness between the cases carrying the TARDBP G298S mutation, we first calculated the pair-wise identity-by-descent (IBD) values between all the 16 cases (8 FALS and 8 SALS). The analysis suggested that the PI_HAT values (proportion of IBD) was 0.52 between F3-1 and F3-2, the two siblings of family 3. In contrast, the PI_HAT between patients from other families were 0.03 ± 0.05, which was far smaller than 0.25, the threshold of recent relationship (second-degree relatives and above) between individuals. To further test whether all the cases carrying the mutation were inherited from the same ancestor, we genotyped the eight microsatellite markers (D1S450, D1S244, D1S2736, D1S1151, D1S2667, D1S489, D1S434, D1S2697) flanking the *TARDBP* gene in the 16 cases carrying the G298S mutation, 92 sporadic ALS cases not carrying the mutation and 65 age-matched neurologically normal individuals (Table 2). These 92 SALS patients were selected from the 782 SALS patients not carrying the G298S mutation, and were derived from different geographical region: 40 from Southern and 52 from Northern China. The 7 of 16 patients shared the 117-286-257-145-246-270 (bps) allele at markers D1S2736-D1S1151-D1S2667-D1S489-D1S434-D1S2697, which was not detected in the 92 non-carrier cases (0/92) (*p* < 0.0001) and 65 age-matched and neurologically normal individuals (0/65) (*p* < 0.0001). The large haplotype spans a 5.8 Mb fragment that includes the *TARDBP* gene. For each marker, the number of alleles observed in the control group and the frequency of the shared alleles were shown in Table 2. In particular, the 286 and 257 bp alleles for the D1S1151 and D1S2667, which were very close (0.3 Mb) to the *TARDBP* gene, were shared by 9 mutation-carriers but were not frequent in the non-carriers (10% and 18%). These data suggest that the G298S mutation may come from a common ancestral chromosome.

**Table 2:** Genotypes of microsatellites flanking TARDBP in the ALS patients carrying the G298S mutation

Marker (Mb)^a^	F1	F2	F3-1	F3-2	F4	F5	F6	F7	S1
D1S450 (−9.585)	**250**/252	251/257	248/**250**	248/**250**	**250**/254	248/254	248/256	**250**/256	254/256
D1S244 (−10.574)	286/**290**	288/**290**	298/300	288/**290**	286/**290**	288/**290**	**290**/294	288/**290**	284/**290**
D1S2736 (−10.615)	118/124	115/**117**	**117**/119	**117**/119	**117**/125	119/121	119/125	**117**/119	**117**/123
TARDBP (−11.083)									
D1S1151 (−11.464)	278/290	**286**/310	260/**286**	260/**286**	282/290	268/290	275/293	278/**286**	268/**286**
D1S2667 (−11.487)	262/268	**257**/265	**257**/259	**257**/259	258/266	261/277	**257**/269	**257**/259	**257**/259
D1S489 (−12.048)	136/144	139/**145**	139/**145**	139/**145**	136/144	141/147	139/**145**	136/138	143/**145**
D1S434 (−12.332)	238/**246**	**246**/248	244/**246**	244/**246**	244/**246**	**246**/254	**246**/256	**246**/248	**246**/248
D1S2697 (−16.419)	**270**/**270**	**270**/**270**	**270**/274	**270**/274	268/**270**	**270**/**270**	**270**/**270**	**270**/**270**	268/**270**
Share the 5.8 Mb haplotype	**No**	**Yes**	**Yes**	**Yes**	**No**	**No**	**No**	**No**	**Yes**

Marker (Mb)^a^	S2	S3	S4	S5	S6	S7	S8	Shared allele^b^	Frequency		
									G298S Cases^c^	Non- G298S Cases^d^	Control^e^
D1S450 (−9.585)	244/**250**	**250**/256	248/**250**	248/**250**	248/**250**	244/**250**	**250**/258	250	0.75	0.28	0.49
D1S244 (−10.574)	286/288	288/**290**	286/**290**	282/**290**	286/**290**	286/**290**	298/300	290	0.81	0.58	0.46
D1S2736 (−10.615)	**117**/119	**117**/119	**117**/119	115/**117**	**117**/119	118/122	**117**/119	117	0.75	0.61	0
TARDBP (−11.083)											
D1S1151 (−11.464)	274/**286**	**286**/292	290/310	252/**286**	290/306	**286**/302	282/290	286	0.56	0.1	0.11
D1S2667 (−11.487)	**257**/265	**257**/265	**257**/271	**257**/259	**257**/265	**257**/269	**257**/271	257	0.81	0.18	0
D1S489 (−12.048)	143/**145**	143/**145**	137/**145**	139/**145**	138/146	139/143	137/**145**	145	0.63	0.33	0
D1S434 (−12.332)	**246**/248	242/**246**	242/**246**	**246**/248	242/**246**	**246**/256	238/**246**	246	1	0.77	0.82
D1S2697 (−16.419)	**270**/**270**	**270**/274	**270**/**270**	**270**/**270**	**270**/**270**	**270**/**270**	268/**270**	270	1	0.95	0.92
Share the 5.8 Mb haplotype	**Yes**	**Yes**	**No**	**Yes**	**No**	**No**	**No**	117-286-257- 145-246-270	7/16	0/92	0/65

Alleles in the haplotype are presented as length of PCR product in base pairs; a Mb, megabases from chromosome 1p telomere (UCSC Genome Browser on Human March 2006 Assembly); b Allele shared by the largest number of patients with the G298S mutation; c Shared allele frequencies in the patients carrying the G298S mutation for each marker (n = 16); d Shared allele frequencies in patients non-carrying the G298S mutation for each marker (n = 92); e Shared allele frequencies in aged matched neurologically normal individuals for each marker (n = 65). Shared alleles were indicated in bold, and the shared haplotype was indicated by a block of gray color. The frequency of the shared hapotype in G298S carriers, non-G298S cases and controls were indicated in blue

### Geographical distribution and clinical features of TARDBP mutations reported worldwide

**Fig. 2:**
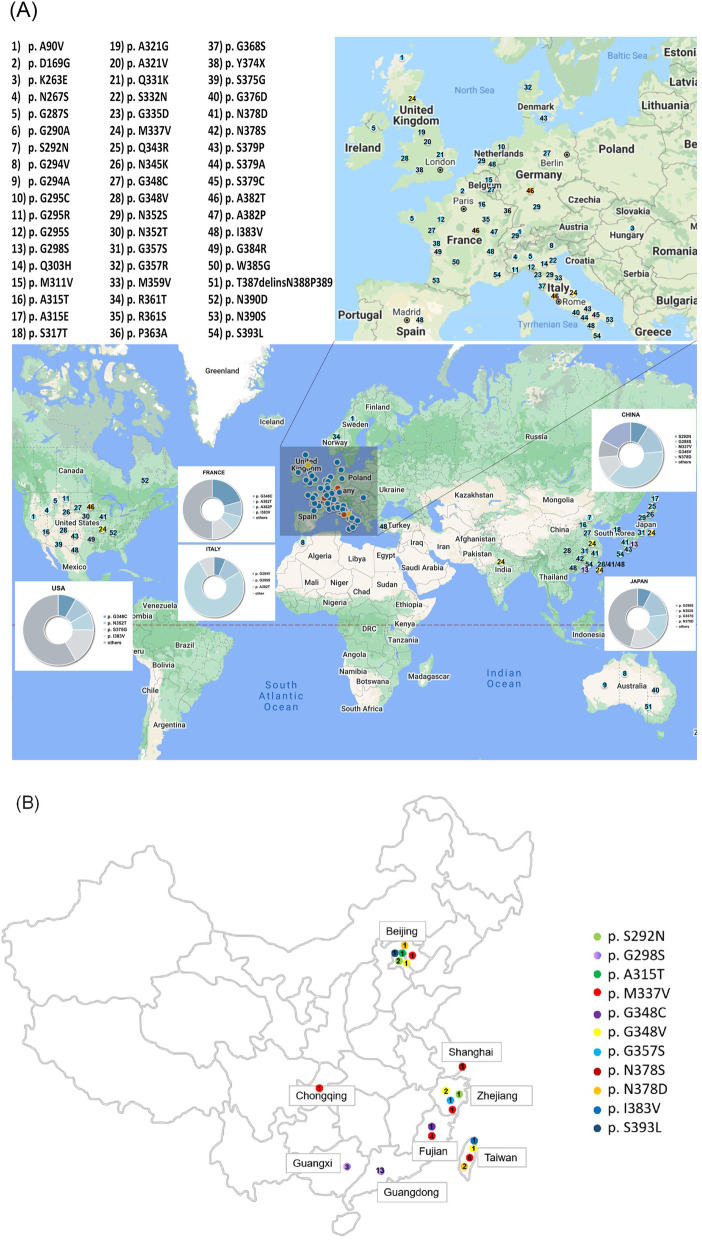
Distribution of TARDBP mutations reported in the world and China. (**A**) Mutations reported in the world. Each mutation was represented for one number. The three prevalent mutations were denoted in dots with different colors: M337V (orange), A382T (yellow) and G298S (purple), and other mutations were denoted in blue dots. The amplified gray square regions represent West Europe which was enlarged on upper right. Five pie charts show the countries in which mutations were more frequently detected. Maps were obtained through My Maps (https://www.google.com/mymaps, Accessed in 05.30.2021). (**B**) Map of China with reported TARDBP mutations. Different mutations were denoted with different colors. The number on the dot represents the number of reported cases

To date, over 50 mutations of *TARDBP* have been described in both familial and sporadic ALS cases (Additional file 1: Table 2). A geographical map of the mutations highlights the qualitative distribution patterns (Fig. 2A). Most reported mutations were detected in countries of North America (US, Canada), Europe (France, Italy, Norway, Germany, Denmark) and Asia (India, Japan, Korea and China). Moreover, mutations were mostly detected in the exon 6 of *TARDBP* and dominantly reported in different geographic regions, such as I383V and S375G in the US, G348C and A382T in France, A382T and G295S in Italy, N352S, G357S and N378D in Japan, and M337V and G298S in China. According to the reported cases, most of the mutations, such as M337V, G348C, N352S and I383V, showed a distribution in multiple ethnicities, while some mutations were detected in specific geographic regions. For example, the A382T mutation was the most frequent mutation in Europe and America but not in Eastern Asians, while G298S was only detected in Eastern Asians. Among the 11 mutations reported in Chinese cases, M337V was most frequently reported in Fujian and Taiwan. In contrast, the G298S mutation was common in Guangdong and Guangxi (Fig. 2B, Table 3).

**Table 3:** Clinical characteristics of the hotspot mutations in TARDBP

Characteristics	G298S	M337V	A382T	G287S	G294V	G295S	G348C	I383V	N352S
Birth place^a^	East Asia	Admixed	Europe and America	Europe and America	Italy	Italy	Admixed	Admixed	Admixed
Family History^b^ (Y/N)	10/10	1/15	78/95	0/9	8/8	4/10	5/4	6/2	11/4
Gender (M/F)^c^	13/9	28/30	122/60	2/0	9/6	2/1	9/6	1/2	1/8
Age at onset	51.3 (8.3)	51.7	52.7	67.5	61.1	60.0	49.9	58.3	57.1
(mean years, SD)		(7.1)	(12.9)	(3.5)	(11.2)	(5.3)	(11.3)	(11.4)	(12.7)
Onset site	L:15; B:6	L:22; B:37	L:141; B:36	L:1; B:1	L:7; B:8	L:3; B:0	L:13; B:2	L:1; L + B:2	L:7; B:0
Disease duration (mean months)	19.3	75.9	57.3	57.5	19.1	32.0	49.1	36.0	63.1
Cognitive impairment	No	Yes	Yes	No	Yes	Yes	No	Yes	No

^a^Birthplace: admixed: the mutation can be detected in both Asian and European patients

^b^Family history: *Y* Yes, *N* No

^c^Gender: *M* male, *F* female

Onset site: *L* limb, *B* bulbar

In demographic and clinical characteristics, except for G287S, most of the mutations were detected in both familial and sporadic cases, and most of the cases displayed onset of limb weakness. However, carriers for M337V mainly developed the disease with a bulbar-onset (bulbar/limb: 37/22) and had the longest survival (75.9 months), while the G298S carriers displayed a limb onset and the shortest survival (19.3 months). At onset age, carriers for three mutations had a later onset (> 60 years): G287S, G294V and G295S (Table 3).

### Clinical characteristics of ALS patients with the TARDBP G298S mutation

The 16 FALS and SALS patients carrying the G298S mutation had similar clinical features (Table 4). The onset age varied considerably, cases S3 and S4 showed early onset at 38 and 39 years old, while F4 had onset at 73 years old. Of all the patients, 11 showed limb-onset, while 5 showed bulbar-onset. Most of the patients displayed fasciculation and signs of dysfunction in both upper motor neuron (UMN) and lower motor neuron (LMN). No signs of dementia were reported in these patients. The severity of neurological functions varies: the scores for ALS functional rating scale-revised (ALSFRS-R) ranged 20–48. Electromyography (EMG) examination was performed in 12 patients, all showed fibrillations, fasciculations and positive sharp waves. Except F1, all the patients had survived no longer than 24 months. In particular, the survival time of S4 and S7 was 7–8 months since onset. Most of the cases did not have histories of drinking, smoking or pesticide exposure. Five patients (F6, F7, S5, S6 and S7) carried another rare variant in the ALS-causing genes (*ALS2*, *SPG11*, *FIG4*, *NEK1*), but all these variants were classified as variant of unknown significance (VUS), according to the ACMG guidelines.

**Table 4:** The demographic and clinical features of the ALS patients carrying the TARDBP G298S mutation

Patient^a^	F1	F2	F3-1	F3-2	F4	F5	F6	F7	S1	S2	S3	S4	S5	S6	S7	S8
Gender	F	F	F	M	F	F	F	M	F	M	F	F	M	M	M	M
Birthplace^b^	GD	GD	GD	GD	GD	GD	GD	GD	GX	GD	GD	GD	GX	GD	GX	GD
Diagnosis level^e^	Def	Def	Def	Def	Def	Prob	Poss	Def	Def	Def	Prob	Def	Poss	Def	Poss	Def
AAO (years)	50	50	62	54	73	49	53	46	43	53	38	39	59	49	52	59
Site of onset^c^	U	L	U	B	U	B	U	B	U	U	U	B	U	U	B	U + L
UMN signs	Yes	Yes	Yes	Yes	Yes	Yes	Yes	Yes	Yes	Yes	Yes	Yes	Yes	Yes	No	Yes
LMN signs	No	Yes	Yes	Yes	Yes	Yes	No	No	Yes	Yes	Yes	Yes	No	Yes	Yes	Yes
Fasciculation	Yes	Yes	Yes	No	Yes	Yes	Yes	Yes	Yes	Yes	Yes	Yes	Yes	Yes	Yes	Yes
ALSFRS-R	48	46	44	NA	40	37	37	45	42	42	NA	NA	NA	46	40	20
Survival (months)	> 48	15	13	20	10	11	20	14	NA	14	NA	< 7	NA	24	8	NA
EMG^d^	NA	Fib Fas PSW	Fib Fas PSW	Fib Fas PSW	Fib Fas PSW	Fib Fas PSW	Fib Fas PSW	Fib Fas PSW	Fib Fas PSW	Fib Fas PSW	NA	Fib Fas PSW	NA	Fib Fas PSW	Fib Fas PSW	NA
Other variants	No	No	No	No	No	No	ALS2 A1550T	SPG11 L1982S	No	No	No	No	FIG4 I220V	NEK1 D1208N	No	No
Smoking	No	No	No	No	No	No	No	No	No	Yes	NA	No	NA	Yes	No	No
Drinking	No	No	No	No	No	No	No	No	No	No	NA	No	NA	No	No	No
Pesticide	No	No	No	No	No	No	No	Yes	No	No	NA	No	NA	Yes	No	No

^a^Patient: F, familial case, S = sporadic case, F3-1 (II-2) and F3-2 (II-3) are the two cases in the family 3; ^b^Birthplace: GD, Guangdong; GX, Guangxi; ^c^Site of onset: *U* upper limb, *L* Lower limb, *B* Bulbar, ^d^EMG: Fib, Fibrillations, Fas, fasciculations, PSW, positive sharp waves; Diagnosis level: Def, definite; Prob, probable; Poss, possible. NA, not available

## Discussion

In this study, we identified G298S as a frequent mutation that were merely detected in patients from the Southern Chinese provinces, and haplotype analysis revealed that inheritance of the G298S mutation was attributable to a founder effect. We also reviewed the *TARDBP* mutations reported worldwide and found that some prevalent mutations were apparently distributed in geographical prevalence and associated with specific clinical features.

Increasing studies have reported the correlation of mutations in ALS genes with specific haplotypes. For *TARDBP* variants, haplotype analyses for the A382T [19, 40], N352S [42] and G294V [43] mutations have been documented. However, these mutations had been mostly reported in Westerners but scarcely reported in Easterners. Before this report, the G298S mutation has only been described in Chinese and Japanese FALS families [44–46]. Our study demonstrates that the high prevalence of the G298S mutation in Southern Chinese patients is due to a founder effect. The analysis of microsatellite markers surrounding the *TARDBP* gene in cases carrying the mutation showed that they were inherited from a common ancestor with the D1S2736-D1S1151-D1S2667-D1S489-D1S434-D1S2697 haplotype. Our study showed that not all the G298S carriers shared the D1S2736-D1S1151-D1S2667-D1S489-D1S434-D1S2697 haplotype, but the frequency of sharing the haplotype (7/16) by the FALS cases was significantly higher than the ALS patients not carrying the mutation (0/92) and general controls (0/65) (*P* < 0.0001). Thus, we assume that the haplotype was not minimal but was strongly associated with the G298S-related ALS. Moreover, our haplotype encloses a reported 94–single-nucleotide polymorphism risk haplotype spanning 663 Kb across the TARDBP locus (between the D1S2736 and D1S2667) on chromosome 1p36.22, which was shared by Sardinia carriers of the A382T mutation [19]. As reported, SOD1 mutations were the most frequent cause for FALS pedigrees in the East-Asian populations, where C9orf72 expansion mutations were rare; furthermore, causative variants were not identified in approximately 40% of FALS pedigrees and TARDBP mutations were not so frequent in the world-wide populations. Thus, the FALS series in this study were particularly different from the previously reported FALS series.

Clinically, no patient with the G298S mutation exhibited overt cognitive impairment in our study, although the TDP-43 protein inclusions being originally identified in FTD cases [47]. However, a limitation of our study is that we were unable to perform postmortem studies to investigate this. Interestingly, as reported here and in the Chinese and Japanese FALS cases, most of the patients carrying this mutation displayed a relatively short survival (< 25 months). These data suggest that G298S is a mutation with unique genetic basis and clinical relevance. However, due to the rarity of the mutation carriers, it remains unclear where was this founder effect originated and transmitted. Population-based studies that would be taken into account biologic and sociocultural factors would help in the understanding of these questions.

The consequences of *TARDBP* mutations on TDP-43 function and neuro-degeneration remain unclear. Studies have suggested that TDP-43 binds and alters RNA metabolism in the cytoplasm through a toxic gain of function whereas the nuclear depletion of TDP-43 could lead to aberrant RNA metabolism through a loss of function [17]. More than 15 mouse models have been reported in which WT and ALS mutant of human TDP-43 are exogenously expressed [48], but the disease relevance of these systems to ALS is uncertain because of the considerable variability in the observed motor phenotypes, and the overexpression of WT as well as mutant TDP-43 leads to a similar motor phenotype [49]. Additionally, the pathological effects of the mutations on neuronal cells remain to be established, although it has been putatively proposed to be related to increased aggregation propensity, enhanced cytoplasmic mislocalization [15]. A recent study using the knock-in mice expressing the M337V and G298S mutations showed that the hemizygous mutations did not influence TDP-43 levels, changed cellular localization in nucleus or interfered with the autoregulation of TDP-43 levels by 2 years of age. In contrast, most homozygous knockin animals display asymmetric denervation of tibialis anterior muscle at 2.5 years of age, demonstrating the dose dependent MN toxicity of the TDP-43 mutant alleles. Moreover, TDP-43 knockin mice showed varying degrees of denervation, consistent with the incomplete penetrance of *TARDBP* mutations in ALS families. These observations raise the question of whether other factors, environmental or genetic, may contribute to TDP-43 pathology and the onset of neurodegeneration. However, such factors have not been documented and could be identified via studies using larger cohorts and more advanced sequencing technologies.

Although the genotype–phenotype correlations of *TARDBP* mutations have been reported, the inter-mutational differences in distribution and clinical features remain elusive. We reviewed the literature-reported mutations and revealed a geographic pattern of the recurrent mutations. Interestingly, the M337V, G348C, N352S and I383V mutations were detected in cases all over the world, whereas the A382T was predominantly detected in populations of European ancestry (especially Sardinian) [28] and American, but not in Asians. The clinical phenotype encompasses patients with bulbar and limb onset and with short and long disease durations. Although most of the cases with mutations displayed limb onset, a bulbar symptoms-dominant onset and the longest disease duration was reported in the carriers for M337V mutation [50]. In contrast, a limb onset and the shortest survival was observed in our carriers for G298S mutation. These data suggest the distinct geographic distribution and clinical features of different mutations, and the differences in the site of onset in patients with different mutations does not seem to explain the differences in survival.

## Conclusions

In conclusion, our study identified G298S as a unique mutation showing founder effect in cases from Southern China. However, the number of cases carrying the mutation were relatively small, and more G298S carriers are needed in the future to gain a clearer genotype–phenotype picture of this rare mutation. In addition, we reviewed the correlation of geographic and clinical features with *TARDBP* mutations reported worldwide, highlighting the mutation hotspots in Western and Eastern countries.

## Supplementary Information

**Additional file 1**. Primers designed for amplifying the microsatellites surrounding the TARDBP gene and all the published mutations in the TARDBP gene.

## Data Availability

Detailed mutational and clinical data of all patients are submitted as Tables 1 and 4. Genotypes of microsatellites flanking TARDBP in the ALS patients carrying the G298S mutation are submitted as Table 2. The primer sequences are shown in Additional file 1. Geography-mutation analyses were based on published reports or databases. The raw sequence data reported in this paper have been deposited in the Genome Sequence Archive [51] in National Genomics Data Center [52], China National Center for Bioinformation/Beijing Institute of Genomics, Chinese Academy of Sciences (GSA-Human: HRA001905) that are publicly accessible at https://ngdc.cncb.ac.cn/gsa-human.

## Abbreviations

ALS: Amyotrophic lateral sclerosis
FTD: Frontotemporal dementia
PCR: Polymerase chain reaction
HRE: Hexanucleotide repeat expansion
WES: Whole-exome sequencing
ACMG/AMP: American College of Medical Genetics and Genomics and the Association for Molecular Pathology
SIFT: Sorting intolerant from tolerant
CADD: Combined annotation-dependent depletion
GERP: Genomic evolutionary rate profiling
UMN: Upper motor neuron
LMN: Lower motor neuron
ALSFRS-R: ALS functional rating scale-revised
EMG: Electromyography
VUS: Variant of unknown significance
