# Supplementary material for: Identification of TARDBP Gly298Ser as a founder mutation for amyotrophic lateral sclerosis in Southern China
Source: BMC Med Genomics. 2022 Aug 5;15:173. doi: 10.1186/s12920-022-01327-4 (PMC9356425; doi:10.1186/s12920-022-01327-4)
Supplement: Supplementary file 1 — Additional file 1. Primers designed for amplifying the microsatellites surrounding the TARDBP gene and all the published mutations in the TARDBP gene. [file 12920_2022_1327_MOESM1_ESM.docx]

**Supplemental Table**

| Supplemental Table 1. Primers designed for amplifying the microsatellites surrounding the TARDBP gene | | | |
| --- | --- | --- | --- |
| **Microsatellite** | **Upper primer** | **Lower primer** | **Distance (bps)** |
| D1S450 | 5'-FAM-GCTCCAATGTCCAAGGG-3' | 5'-GGGTACTCAGATGGCTGGT-3' | 243-267 |
| D1S244 | 5'-TAMRA-GAGCAGCACCGTACAAAT-3' | 5'-AGCTCCGCTCCCTGTAAT-3' | 285-296 |
| D1S2736 | 5'-FAM-CCTCCAGGGTATTCTTGG-3' | 5'-TTTTTGAGGTGTGAGAGCAG-3' | 122-132 |
| D1S2667 | 5'-FAM-AGCTTCAGGTCTGGGGGACA-3' | 5'-CTGGGTTTACCCTCCAGCGA-3' | 224-272 |
| D1S1151 | 5'-TAMRA-TTGCAGTGAGCCGATATCG-3' | 5'-TCCCCATTTAGATTGAAGGG-3' | 239-332 |
| D1S434 | 5'-FAM-AGCTAATTTACATTACCCAAAAAGA-3' | 5'-GCAGGTGGCACAGTGA-3' | 240-252 |
| D1S489 | 5'-FAM-AGCCAGACCAAGTCTCAACA-3' | 5'-ACAAAATGATGGGGTTATGG-3' | 141-153 |
| D1S2697 | 5'-TAMRA-GGGCCACAGAGTGAGAC-3' | 5'-GGCAGAGGTGGTTAAGG-3' | 273-281 |

Supplemental Table 2. All the published mutations in the TARDBP gene

| **DNA mutation** | **Protein change** | **Exon** | **Clinvar**  **Classification** | **F**  **ALS** | **S**  **ALS** | **Site of onset** | **Other**  **phenotype** | **Origin** | **References** |
| --- | --- | --- | --- | --- | --- | --- | --- | --- | --- |
| c.269C>T | p. A90V | 3 | VUS | 4 | 1 | B, S | - | UK, USA, Swiss, Sweden | [1][2][3] |
| c.506A>G | p. D169G | 4 | PAT (1), VUS (1) | - | 1 | S | - | France | [4] |
| c.787A>G | p. K263E | 6 | PAT | - | - | Non-  MND | FTD | Hungary | [5] |
| c.800A>G | p. N267S | 6 | PAT | - | 2 | S | FTD, PD, CBS [6-12] | Italy, USA | [13][14] |
| c.859G>A | p. G287S | 6 | PAT (1),LP (1),VUS (1) | - | 9 | B, S | - | Italy, USA, France, UK | [13][15][4][16][1][17][18] |
| c.869G>C | p. G290A | 6 | VUS | 1 | - | B, S | - | Europe | [19] |
| c.875G>A | p. S292N | 6 | NA | 1 | 2 | B, S | - | China | [20][21] |
| c.881G>T | p. G294V | 6 | PAT | 8 | 9 | B, S | Dementia | Australia, Italy, Morocco | [22][14][23][24][25][26][27]. |
| c.881G>C | p. G294A | 6 | PAT | - | 1 | S | - | Australia | [28] |
| c.883G>T | p. G295C | 6 | NA | 1 | - | NA | - | Netherlands | [29] |
| c.883G>C | p. G295R | 6 | NA | - | 2 | B | - | Italy, USA | [13][30] |
| c.883G>A | p. G295S | 6 | PAT | 4 | 10 | B, S | FTD/Dementia [31] | Italy, France | [23][26][32]  [13][33][34] |
| c.892G>A | p. G298S | 6 | PAT | 3 | 2 | B, S | - | China, Japan | [19][35][36][37] |
| c.909A>C | p. Q303H | 6 | NA | - | 1 | S | - | Italy | [14] |
| c.931A>G | p. M311V | 6 | VUS | 1 | - | B | - | Belgium | [38] |
| c.943G>A | p. A315T | 6 | PAT | 3 | - | S | - | Europe, France, USA, China | [4][39][30]. |
| c.944C>A | p. A315E | 6 | NA | 1 | - | S | PD | Japan | [40] |
| c.950G>C | p. S317T | 6 | NA | - | 1 | S | - | Korean | [41] |
| c.962C>G | p. A321G | 6 | NA | - | 1 | S | - | England | [42] |

Supplemental Table 2. All the published mutations in the TARDBP gene (Continued)

| **DNA mutation** | **Protein change** | **Exon** | **Clinvar**  **Classification** | **F**  **ALS** | **S**  **ALS** | **Site of onset** | **Other**  **phenotype** | **Origin** | **References** |
| --- | --- | --- | --- | --- | --- | --- | --- | --- | --- |
| c. 962 C>T | p. A321V | 6 | NA | - | 1 | S | - | England | [17] |
| c.991C>A | p. Q331K | 6 | PAT | - | 1 | S | - | England | [28] |
| c.995G>A | p. S332 N | 6 | NA | 1 | - | S | - | Italy | [13] |
| c.1004G>A | p. G335D | 6 | NA | - | 1 | S | - | Italy | [13] |
| c.1009A>G | p. M337V | 6 | PAT | 19 | 1 | B, S | - | China, England, Italy,  Japan, Indian, USA | [43][44][45][46][17][1]  [47][28][13][48][49] |
| c.1028A>G | p. Q343R | 6 | PAT | 1 | - | B | - | Japan | [50] |
| c.1035C>A | p. N345K | 6 | PAT | 3 | - | B, S | - | USA, Japan | [49][30][51] |
| c.1042G>T | p. G348C | 6 | PAT (1), LP (1) | 5 | 4 | B, S | - | France, Belgium, Germany, USA, China | [4][52][53][26][54][30] |
| c.1043G>T | p. G348V | 6 | PAT | 4 | 2 | S | - | China, England, USA | [55][21][17][1][56][57] |
| c.1055A>G | p. N352S | 6 | PAT | 11 | 4 | S | PMA | Netherlands, Germany, Japan, Swiss, Italy | [29,58][54][59][60][61][62][3][63] |
| c.1055 A>C | p. N352T | 6 | NA | 2 | - | S | - | USA | [30] |
| c.1069G>A | p. G357S | 6 | NA | - | 2 | S | - | Japan, China | [61] |
| c.1069G>C | p. G357R | 6 | NA | 1 | - | B | - | Denmark | [64] |
| c.1075A>G | p. M359V | 6 | NA | - | - | Non-  MND | FTD | Italy | [34] |
| c.1082G>C | p. R361T | 6 | NA | 2 | - | S | FTD | Norway | [64]. |
| c.1083G>T | p. R361S | 6 | NA | - | 1 | S | - | France | [4]. |
| c.1086G>C | p. P363A | 6 | NA | - | 1 | S | - | France | [53]. |
| c.1102G>A | p. G368S | 6 | NA | - | 1 | B | - | Italy | [65]. |

Supplemental Table 2. All the published mutations in the TARDBP gene (Continued)

| **DNA mutation** | **Protein change** | **Exon** | **Clinvar**  **Classification** | **F**  **ALS** | **S**  **ALS** | **Site of onset** | **Other**  **phenotype** | **Origin** | **References** |
| --- | --- | --- | --- | --- | --- | --- | --- | --- | --- |
| c. 1120_1121insA | p. Y374X | 6 | NA | 1 | 1 | S | - | France, UK | [53][66] |
| c.1123A>G | p. S375G | 6 | NA | 1 | 1 | S | - | USA | [67][15] |
| c.1127G/A | p. G376D | 6 | NA | 2 | - | S | - | Australia, Italy | [68][25] |
| c.1132>G | p. N378D | 6 | VUS | 4 | - | B, S | - | USA, Taiwan Japan, China | [30][46][69] |
| c.1133>G | p. N378S | 6 | NA | - | 1 | S | - | China | [70] |
| c.1135T>C | p. S379P | 6 | NA | 4 | - | B, S | - | Italy, Japan Denmark, USA | [13][69][64][30] |
| c.1135T>G | p. S379A | 6 | NA | - | 1 | S | - | Italy | [71] |
| c.1136C>G | p. S379C | 6 | NA | - | 1 | S | - | Italy | [13] |
| c.1144G>A | p. A382T | 6 | LP | 65 | 79 | B, S | FTD/PD | Italy, France, USA, Germany | [74][33][75][16][76][13][77][4][57][78] |
| c.1144G>C | p. A382P | 6 | NA | - | 2 | S | Sensory | France | [53][79] |
| c.1147A>G | p. I383V | 6 | PAT (1),  LP (1) | 6 | 2 | B, S | FTD | USA, France, Netherlands,  Turkey, Italy, China | [49][30][82]  [29,58][83][14] |
| c.1150G>C | p. G384R | 6 | VUS | 2 | - | S | - | France, USA | [52][30] |
| c.1153T>G | p. W385G | 6 | PAT | 1 | - | S | - | France | [52] |
| c.1158_1159delAT  insCACCAACC | p. T387del  insN388P389 | 6 | NA | 1 | - | S | - | Australia | [68] |
| c.1168A>G | p. N390D | 6 | NA | 1 | 1 | S | - | USA, Canada | [30][4] |
| c.1169A>G | p. N390S | 6 | NA | - | 3 | B, S | - | France, Italy | [4][14] |
| c.1178C>T | p. S393L | 6 | NA | 3 | 1 | S | - | France, Italy, Japan, China | [84][85][69][13] |

MND, motor neuron disease.”-” presents not have been reported. ”B” presents Bulbar, ”S” presents Spinal. ”Non-MND” presents No signs of MND.

**References**

1. Mehta PR, Jones AR, Opie-Martin S, Shatunov A, Iacoangeli A, Al Khleifat A, et al. Younger age of onset in familial amyotrophic lateral sclerosis is a result of pathogenic gene variants, rather than ascertainment bias. J Neurol Neurosurg Psychiatry. 2019;90:268-71.
2. Winton MJ, Van Deerlin VM, Kwong LK, Yuan W, Wood EM, Yu CE, et al. A90V TDP-43 variant results in the aberrant localization of TDP-43 in vitro. FEBS Lett. 2008;582:2252-6.
3. Czell D, Andersen PM, Morita M, Neuwirth C, Perren F, Weber M. Phenotypes in Swiss patients with familial ALS carrying TARDBP mutations. Neurodegener Dis. 2013;12:150-5.
4. Kabashi E, Valdmanis PN, Dion P, Spiegelman D, McConkey BJ, Vande Velde C, et al. TARDBP mutations in individuals with sporadic and familial amyotrophic lateral sclerosis. Nat Genet. 2008;40:572-4.
5. Stochl J, Hagtvet KA, Brozová H, Klempír J, Roth J, Růzicka E. Handedness does not predict side of onset of motor symptoms in Parkinson's disease. Mov Disord. 2009;24:1836-9.
6. Borroni B, Archetti S, Del Bo R, Papetti A, Buratti E, Bonvicini C, et al. TARDBP mutations in frontotemporal lobar degeneration: frequency, clinical features, and disease course. Rejuvenation Res. 2010;13:509-17.
7. Borroni B, Bonvicini C, Alberici A, Buratti E, Agosti C, Archetti S, et al. Mutation within TARDBP leads to frontotemporal dementia without motor neuron disease. Hum Mutat. 2009;30:E974-83.
8. Origone P, Accardo J, Verdiani S, Lamp M, Arnaldi D, Bellone E, et al. Neuroimaging features in C9orf72 and TARDBP double mutation with FTD phenotype. Neurocase. 2015;21:529-34.
9. Fernández MV, Kim JH, Budde JP, Black K, Medvedeva A, Saef B, et al. Analysis of neurodegenerative Mendelian genes in clinically diagnosed Alzheimer Disease. PLoS Genet. 2017;13:e1007045.
10. Gagliardi M, Arabia G, Nisticò R, Iannello G, Procopio R, Manfredini L, et al. Mutational analysis of TARDBP gene in patients affected by Parkinson's disease from Calabria. J Neurol Sci. 2018;390:209-11.
11. Rayaprolu S, Fujioka S, Traynor S, Soto-Ortolaza AI, Petrucelli L, Dickson DW, et al. TARDBP mutations in Parkinson's disease. Parkinsonism Relat Disord. 2013;19:312-5.
12. Huey ED, Ferrari R, Moreno JH, Jensen C, Morris CM, Potocnik F, et al. FUS and TDP43 genetic variability in FTD and CBS. Neurobiol Aging. 2012;33:1016.e9-17.
13. Corrado L, Ratti A, Gellera C, Buratti E, Castellotti B, Carlomagno Y, et al. High frequency of TARDBP gene mutations in Italian patients with amyotrophic lateral sclerosis. Hum Mutat. 2009;30:688-94.
14. Lattante S, Conte A, Zollino M, Luigetti M, Del Grande A, Marangi G, et al. Contribution of major amyotrophic lateral sclerosis genes to the etiology of sporadic disease. Neurology. 2012;79:66-72.
15. Cady J, Allred P, Bali T, Pestronk A, Goate A, Miller TM, et al. Amyotrophic lateral sclerosis onset is influenced by the burden of rare variants in known amyotrophic lateral sclerosis genes. Ann Neurol. 2015;77:100-13.
16. Ticozzi N, Tiloca C, Mencacci NE, Morelli C, Doretti A, Rusconi D, et al. Oligoclonal bands in the cerebrospinal fluid of amyotrophic lateral sclerosis patients with disease-associated mutations. J Neurol. 2013;260:85-92.
17. Kirby J, Goodall EF, Smith W, Highley JR, Masanzu R, Hartley JA, et al. Broad clinical phenotypes associated with TAR-DNA binding protein (TARDBP) mutations in amyotrophic lateral sclerosis. Neurogenetics. 2010;11:217-25.
18. Ranganathan R, Haque S, Coley K, Shepheard S, Cooper-Knock J, Kirby J. Multifaceted Genes in Amyotrophic Lateral Sclerosis-Frontotemporal Dementia. Front Neurosci. 2020;14:684.
19. Van Deerlin VM, Leverenz JB, Bekris LM, Bird TD, Yuan W, Elman LB, et al. TARDBP mutations in amyotrophic lateral sclerosis with TDP-43 neuropathology: a genetic and histopathological analysis. Lancet Neurol. 2008;7:409-16.
20. Xiong HL, Wang JY, Sun YM, Wu JJ, Chen Y, Qiao K, et al. Association between novel TARDBP mutations and Chinese patients with amyotrophic lateral sclerosis. BMC Med Genet. 2010;11:8.
21. Zou ZY, Peng Y, Wang XN, Liu MS, Li XG, Cui LY. Screening of the TARDBP gene in familial and sporadic amyotrophic lateral sclerosis patients of Chinese origin. Neurobiol Aging. 2012; 33:2229.e11-2229.e18.
22. Williams KL, Durnall JC, Thoeng AD, Warraich ST, Nicholson GA, Blair IP. A novel TARDBP mutation in an Australian amyotrophic lateral sclerosis kindred. J Neurol Neurosurg Psychiatry. 2009;80:1286-8.
23. Piaceri I, Del Mastio M, Tedde A, Bagnoli S, Latorraca S, Massaro F, et al. Clinical heterogeneity in Italian patients with amyotrophic lateral sclerosis. Clin Genet. 2012;82:83-7.
24. Corrado L, Pensato V, Croce R, Di Pierro A, Mellone S, Dalla Bella E, et al. The first case of the TARDBP p.G294V mutation in a homozygous state: is a single pathogenic allele sufficient to cause ALS? Amyotroph Lateral Scler Frontotemporal Degener. 2020;21:273-9.

25. Conforti FL, Sproviero W, Simone IL, Mazzei R, Valentino P, Ungaro C, et al. TARDBP gene mutations in south Italian patients with amyotrophic lateral sclerosis. J Neurol Neurosurg Psychiatry. 2011;82:587-8.

26. Del Bo R, Ghezzi S, Corti S, Pandolfo M, Ranieri M, Santoro D, et al. TARDBP (TDP-43) sequence analysis in patients with familial and sporadic ALS: identification of two novel mutations. Eur J Neurol.2009;16:727-32.

27. Guennoc AM, Heuze-Vourc'h N, Gordon PH, Courty Y, Vourc'h P, Andres CR, et al. Benign lower limb amyotrophy due to TARDBP mutation or post-polio syndrome? Amyotroph Lateral Scler Frontotemporal Degener. 2013;14:476-8.

28. Sreedharan J, Blair IP, Tripathi VB, Hu X, Vance C, Rogelj B, et al. TDP-43 mutations in familial and sporadic amyotrophic lateral sclerosis. Science. 2008;319:1668-72.

29. van Blitterswijk M, van Es MA, Hennekam EA, Dooijes D, van Rheenen W, Medic J, et al. Evidence for an oligogenic basis of amyotrophic lateral sclerosis. Hum Mol Genet. 2012;21:3776-84.

30. Ticozzi N, LeClerc AL, van Blitterswijk M, Keagle P, McKenna-Yasek DM, Sapp PC, et al. Mutational analysis of TARDBP in neurodegenerative diseases. Neurobiol Aging. 2011;32:2096-9.

31. Benajiba L, Le Ber I, Camuzat A, Lacoste M, Thomas-Anterion C, Couratier P, et al. TARDBP mutations in motoneuron disease with frontotemporal lobar degeneration. Ann Neurol. 2009;65:470-3.

32. Lattante S, Rouleau GA, Kabashi E. TARDBP and FUS Mutations Associated with Amyotrophic Lateral Sclerosis: Summary and Update. Hum Mutat. 2013;34:812-6.

33. Borghero G, Pugliatti M, Marrosu F, Marrosu MG, Murru MR, Floris G, et al. Genetic architecture of ALS in Sardinia. Neurobiol Aging. 2014;35:2882.e7-2882.e12.

34. Kaivorinne AL, Moilanen V, Kervinen M, Renton AE, Traynor BJ, Majamaa K, et al. Novel TARDBP sequence variant and C9ORF72 repeat expansion in a family with frontotemporal dementia. Alzheimer Dis Assoc Disord. 2014;28:190-3.

35. Nozaki I, Arai M, Takahashi K, Hamaguchi T, Yoshikawa H, Muroishi T, et al. Familial ALS with G298S mutation in TARDBP: a comparison of CSF tau protein levels with those in sporadic ALS. Intern Med. 2010;49:1209-12.

36. Pang SY, Hsu JS, Teo KC, Li Y, Kung MHW, Cheah KSE, et al. Burden of rare variants in ALS genes influences survival in familial and sporadic ALS. Neurobiol Aging. 2017;58:238.e9-238.e15.

37. Lin J, Chen W, Huang P, Xie Y, Zheng M, Yao X. The distinct manifestation of young-onset amyotrophic lateral sclerosis in China. Amyotroph Lateral Scler Frontotemporal Degener. 2021;22:30-7.

38. Lemmens R, Race V, Hersmus N, Matthijs G, Van Den Bosch L, Van Damme P, et al. TDP-43 M311V mutation in familial amyotrophic lateral sclerosis. J Neurol Neurosurg Psychiatry. 2009;80:354-5.

39. Gitcho MA, Baloh RH, Chakraverty S, Mayo K, Norton JB, Levitch D, et al. TDP-43 A315T mutation in familial motor neuron disease.

Ann Neurol. 2008;63:535-8.

40. Fujita Y, Ikeda M, Yanagisawa T, Senoo Y, Okamoto K. Different clinical and neuropathologic phenotypes of familial ALS with A315E TARDBP mutation. Neurology. 2011;77:1427-31.

41. Kim HJ, Oh KW, Kwon MJ, Oh SI, Park JS, Kim YE, et al. Identification of mutations in Korean patients with amyotrophic lateral sclerosis using multigene panel testing. Neurobiol Aging. 2016;37:209.e9-209.e16.

42. Baumer D, Parkinson N, Talbot K. TARDBP in amyotrophic lateral sclerosis: identification of a novel variant but absence of copy number variation. J Neurol Neurosurg Psychiatry. 2009;80:1283-5.

43. Liu ZJ, Lin HX, Wei Q, Zhang QJ, Chen CX, Tao QQ, et al. Genetic Spectrum and Variability in Chinese Patients with Amyotrophic Lateral Sclerosis. Aging Dis. 2019;10:1199-206.

44. Ju X, Liu W, Li X, Liu N, Zhang N, Liu T, et al. Two distinct clinical features and cognitive impairment in amyotrophic lateral sclerosis patients with TARDBP gene mutations in the Chinese population. Neurobiol Aging. 2016;38:216.e1-216.e6.

45. Xu GR, Hu W, Zhan LL, Wang C, Xu LQ, Lin MT, et al. High frequency of the TARDBP p.M337 V mutation among south-eastern Chinese patients with familial amyotrophic lateral sclerosis. BMC Neurol. 2018;18.

46. Tsai CP, Soong BW, Lin KP, Tu PH, Lin JL, Lee YC. FUS, TARDBP, and SOD1 mutations in a Taiwanese cohort with familial ALS. Neurobiol Aging. 2011;32: 553.e13-21.

47. Narain P, Pandey A, Gupta S, Gomes J, Bhatia R, Vivekanandan P. Targeted next-generation sequencing reveals novel and rare variants in Indian patients with amyotrophic lateral sclerosis. Neurobiol Aging. 2018;71:265.e9-265.e14.

48. Tamaoka A, Arai M, Itokawa M, Arai T, Hasegawa M, Tsuchiya K, et al. TDP-43 M337V mutation in familial amyotrophic lateral sclerosis in Japan. Intern Med. 2010;49:331-4.

49. Rutherford NJ, Zhang YJ, Baker M, Gass JM, Finch NA, Xu YF, et al. Novel mutations in TARDBP (TDP-43) in patients with familial amyotrophic lateral sclerosis. PLoS Genet. 2008;4:e1000193.

50. Yokoseki A, Shiga A, Tan CF, Tagawa A, Kaneko H, Koyama A, et al. TDP-43 mutation in familial amyotrophic lateral sclerosis. Ann

Neurol. 2008;63:538-42.

51. Takeda T, Iijima M, Shimizu Y, Yoshizawa H, Miyashiro M, Onizuka H, et al. p.N345K mutation in TARDBP in a patient with familial amyotrophic lateral sclerosis: An autopsy case. Neuropathology. 2019;39:286-93.

52. Millecamps S, Salachas F, Cazeneuve C, Gordon P, Bricka B, Camuzat A, et al. SOD1, ANG, VAPB, TARDBP, and FUS mutations in familial amyotrophic lateral sclerosis: genotype-phenotype correlations. J Med Genet. 2010;47:554-60.

53. Daoud H, Valdmanis PN, Kabashi E, Dion P, Dupré N, Camu W, et al. Contribution of TARDBP mutations to sporadic amyotrophic lateral sclerosis. J Med Genet. 2009;46:112-4.

54. Kühnlein P, Sperfeld AD, Vanmassenhove B, Van Deerlin V, Lee VM, Trojanowski JQ, et al. Two German kindreds with familial amyotrophic lateral sclerosis due to TARDBP mutations. Arch Neurol. 2008;65:1185-9.

55. Liu ZJ, Lin HX, Liu GL, Tao QQ, Ni W, Xiao BG, et al. The investigation of genetic and clinical features in Chinese patients with juvenile amyotrophic lateral sclerosis. Clin Genet. 2017;92:267-73.

56. Morgan S, Shatunov A, Sproviero W, Jones AR, Shoai M, Hughes D, et al. A comprehensive analysis of rare genetic variation in amyotrophic lateral sclerosis in the UK. Brain. 2017;140:1611-8.

57. Brown JA, Min J, Staropoli JF, Collin E, Bi S, Feng X, et al. SOD1, ANG, TARDBP and FUS mutations in amyotrophic lateral sclerosis: a United States clinical testing lab experience. Amyotroph Lateral Scler. 2012;13:217-22.

58. van Blitterswijk M, Vlam L, van Es MA, van der Pol WL, Hennekam EA, Dooijes D, et al. Genetic overlap between apparently sporadic motor neuron diseases. PLoS One. 2012;7:e48983.

59. Brenner D, Yilmaz R, Müller K, Grehl T, Petri S, Meyer T, et al. Hot-spot KIF5A mutations cause familial ALS. Brain. 2018;141:688-97.

60. Kamada M, Maruyama H, Tanaka E, Morino H, Wate R, Ito H, et al. Screening for TARDBP mutations in Japanese familial amyotrophic lateral sclerosis. J Neurol Sci. 2009;284:69-71.

61. Iida A, Kamei T, Sano M, Oshima S, Tokuda T, Nakamura Y, et al. Large-scale screening of TARDBP mutation in amyotrophic lateral sclerosis in Japanese. Neurobiol Aging. 2012;33:786-90.

62. Homma T, Nagaoka U, Kawata A, Mochizuki Y, Kawakami H, Maruyama H, et al. Neuropathological features of Japanese familial amyotrophic lateral sclerosis with p.N352S mutation in TARDBP. Neuropathol Appl Neurobiol. 2014;40:231-6.

63. Trojsi F, Caiazzo G, Corbo D, Piccirillo G, Cristillo V, Femiano C, et al. Microstructural changes across different clinical milestones of disease in amyotrophic lateral sclerosis. PLoS One. 2015;10:e0119045.

64. Chiang HH, Andersen PM, Tysnes OB, Gredal O, Christensen PB, Graff C. Novel TARDBP mutations in Nordic ALS patients. J Hum Genet. 2012;57:316-9.

65. De Marco G, Lupino E, Calvo A, Moglia C, Buccinnà B, Grifoni S, et al. Cytoplasmic accumulation of TDP-43 in circulating lymphomonocytes of ALS patients with and without TARDBP mutations. Acta Neuropathol. 2011;121:611-22.

66. King A, Troakes C, Smith B, Nolan M, Curran O, Vance C, et al. ALS-FUS pathology revisited: singleton FUS mutations and an unusual case with both a FUS and TARDBP mutation. Acta Neuropathol Commun. 2015;3:62.

67. Newell K, Paron F, Mompean M, Murrell J, Salis E, Stuani C, et al. Dysregulation of TDP-43 intracellular localization and early onset ALS are associated with a TARDBP S375G variant. Brain Pathol. 2019;29:397-413.

68. Solski JA, Yang S, Nicholson GA, Luquin N, Williams KL, Fernando R, et al. A novel TARDBP insertion/deletion mutation in the flail arm variant of amyotrophic lateral sclerosis. Amyotroph Lateral Scler. 2012;13:465-70.

69. Watanabe S, Kaneko K, Yamanaka K. Accelerated disease onset with stabilized familial amyotrophic lateral sclerosis (ALS)-linked mutant TDP-43 proteins. J Biol Chem. 2013;288:3641-54.

70. Huang R, Fang DF, Ma MY, Guo XY, Zhao B, Zeng Y, et al. TARDBP gene mutations among Chinese patients with sporadic amyotrophic lateral sclerosis. Neurobiol Aging. 2012;33:1015.e1-6.

71. Sprovieri T, Ungaro C, Perrone B, Naimo GD, Spataro R, Cavallaro S, et al. A novel S379A TARDBP mutation associated to late-onset sporadic ALS. Neurol Sci. 2019;40:2111-8.

72. Quadri M, Cossu G, Saddi V, Simons EJ, Murgia D, Melis M, et al. Broadening the phenotype of TARDBP mutations: the TARDBP Ala382Thr mutation and Parkinson's disease in Sardinia. Neurogenetics. 2011;12:203-9.

73. Blauwendraat C, Wilke C, Simón-Sánchez J, Jansen IE, Reifschneider A, Capell A, et al. The wide genetic landscape of clinical frontotemporal dementia: systematic combined sequencing of 121 consecutive subjects. Genet Med. 2018;20:240-9.

74. Chiò A, Borghero G, Pugliatti M, Ticca A, Calvo A, Moglia C, et al. Large proportion of amyotrophic lateral sclerosis cases in Sardinia due to a single founder mutation of the TARDBP gene. Arch Neurol. 2011;68:594-8.

75. Floris G, Borghero G, Cannas A, Di Stefano F, Murru MR, Corongiu D, et al. Clinical phenotypes and radiological findings in frontotemporal dementia related to TARDBP mutations. J Neurol. 2015;262:375-84.

76. Mandich P, Mantero V, Verdiani S, Gotta F, Caponnetto C, Bellone E, et al. Complexities of Genetic Counseling for ALS: A Case of Two Siblings with Discordant Genetic Test Results. J Genet Couns. 2015;24:553-7.

77. Orrù S, Manolakos E, Orrù N, Kokotas H, Mascia V, Carcassi C, et al. High frequency of the TARDBP p.Ala382Thr mutation in Sardinian patients with amyotrophic lateral sclerosis. Clin Genet. 2012;81:172-8.

78. Synofzik M, Born C, Rominger A, Lummel N, Schöls L, Biskup S, et al. Targeted high-throughput sequencing identifies a TARDBP mutation as a cause of early-onset FTD without motor neuron disease. Neurobiol Aging. 2014;35:1212.e1-5.

79. Camdessanché JP, Belzil VV, Jousserand G, Rouleau GA, Créac'h C, Convers P, et al. Sensory and motor neuronopathy in a patient with the A382P TDP-43 mutation. Orphanet J Rare Dis. 2011;6:4.

80. Takada LT, Bahia VS, Guimarães HC, Costa TV, Vale TC, Rodriguez RD, et al. GRN and MAPT Mutations in 2 Frontotemporal Dementia Research Centers in Brazil. Alzheimer Dis Assoc Disord. 2016;30:310-7.

81. Ramos EM, Koros C, Dokuru DR, Van Berlo V, Kroupis C, Wojta K, et al. Frontotemporal dementia spectrum: first genetic screen in a Greek cohort. Neurobiol Aging. 2019;75:224.e1-224.e8.

82. Caroppo P, Camuzat A, Guillot-Noel L, Thomas-Antérion C, Couratier P, Wong TH, et al. Defining the spectrum of frontotemporal dementias associated with TARDBP mutations. Neurol Genet. 2016;2:e80.

83. Özoğuz A, Uyan Ö, Birdal G, Iskender C, Kartal E, Lahut S, et al. The distinct genetic pattern of ALS in Turkey and novel mutations. Neurobiol Aging. 2015;36:1764.e9-1764.e18.

84. Praline J, Vourc'h P, Guennoc AM, Veyrat-Durebex C, Corcia P. Co-occurrence of progressive anarthria with an S393L TARDBP mutation and ALS within a family. Amyotroph Lateral Scler. 2012;13:155-7.

85. Origone P, Caponnetto C, Bandettini Di Poggio M, Ghiglione E, Bellone E, Ferrandes G, et al. Enlarging clinical spectrum of FALS with TARDBP gene mutations: S393L variant in an Italian family showing phenotypic variability and relevance for genetic counselling. Amyotroph Lateral Scler. 2010;11:223-7.
